# LAMA-1: A Cerebroside Isolated from the Deep-Sea-Derived Fungus *Penicillium chrysogenum* ^†^

**DOI:** 10.3390/metabo10020075

**Published:** 2020-02-20

**Authors:** Samah O. Alshehri, Rania T. Malatani, Hanin A. Bogari, Ahmad O. Noor, Amany K. Ibrahim, Sameh S. Elhady, Reda F. A. Abdelhameed

**Affiliations:** 1 Department of Pharmacy Practice, Faculty of Pharmacy, King Abdulaziz University, Jeddah 21589, Saudi Arabia; salshehri1@kau.edu.sa (S.O.A.); rmalatani@kau.edu.sa (R.T.M.); hbogari@kau.edu.sa (H.A.B.); aonoor@kau.edu.sa (A.O.N.); 2 Department of Pharmacognosy, Faculty of Pharmacy, Suez Canal University, Ismailia 41522, Egypt; am_kamal66@yahoo.com; 3 Department of Natural Products, Faculty of Pharmacy, King Abdulaziz University, Jeddah 21589, Saudi Arabia; 4 Department of Pharmacognosy, Faculty of Pharmacy, Port Said University, Port Said 42526, Egypt

**Keywords:** deep-sea sediment, *Penicillium* sp., cerebroside molecular species, cytotoxic assay

## Abstract

Chemical investigation of the ethyl acetate extract of *Penicillium chrysogenum* strain S003, a fungus isolated from Red Sea deep sediment, led to the isolation of a cerebroside molecular species LAMA (**1**) along with three other known compounds, ergosterol (**2**), epidioxyergosterol (**3**), and kojic acid (**4**). The structures of the isolated compounds were elucidated by interpretation of spectral data, including detailed 1D and 2D NMR (One and two dimensional Nuclear Magnetic Resonance) and mass spectrometry. The cytotoxic activities of isolated compounds **1**–**4** against five human carcinoma cells were evaluated using sulforhodamine B (SRB) assay. Compounds **2** and **3** displayed promising cytotoxic profiles against lung cancer (A-549), prostate (DU-145), breast adenocarcinoma (MCF-7), and hepatocellular (HepG2) cell lines, with IC_50_ values of 21.26, 19.3; 1.50, 6.10; 16.95, 13.6; and 2.89, 3.07 µM, respectively, while they were inactive against HeLa cells. Compounds **1** and **4** showed weak cytotoxic profiles against all cell lines under investigation.

## 1. Introduction

Marine-derived fungi are recognized as a great source of potentially useful natural products [1,2]. Since the late 1990s, hundreds of novel compounds have been isolated and characterized from marine habitats annually [3], in stark contrast to their terrestrial counterparts. The most explored fungal isolates by the natural products chemists are the *Penicillium* species, which are considered a major source for drug discovery [4,5,6,7,8]. Currently, as reported in many recent reviews [9,10,11,12,13], the *Penicillium* genus still represents a major producer of diverse biologically active metabolites.

The *Penicillium* genus and its different spp. are fast-growing, salt-tolerant, and can easily be obtained and subcultured from various substrates, so the high number of reported metabolites could be justified by this fact. This has encouraged many researchers to study variable *Penicillium* species isolated from different habitats [6]. Their extensive investigation is concerned with the isolation, characterization, and bioactivity assessment of the obtained secondary metabolites. Among the significant reported activities are antibacterial [14,15,16,17], cytotoxic, and anticancer [16,17,18,19,20,21] activities.

In the course of our ongoing efforts to isolate and identify drug leads from marine metabolites [22,23,24,25,26,27], we have investigated the extract of the Red Sea deep-sediment isolated fungus *Penicillium chrysogenum* strain S003.

Here, we report the isolation, structure elucidation, and cytotoxic profiles of the purified compounds, including a cerebroside molecular species (**1**), together with the known reported compounds ergosterol (**2**) [28,29,30], epidioxyergosterol (**3**) [30,31], and kojic acid (**4**) [32,33,34,35].

## 2. Results and Discussion

### 2.1. Isolation and Purification of Compounds ***1**–**4***

The chromatographic fractionation of extracts from the broth and mycelia of fungus *Penicillium chrysogenum* strain S003 using silica gel and Sephadex LH-20 column chromatography resulted in the isolation of four separate compounds (**1**–**4**). Compound **1** was assigned as a cerebroside molecular species, and the name LAMA was assigned to this compound.

### 2.2. Structural Elucidation of Compounds ***1**–**4***

**Figure 1:**
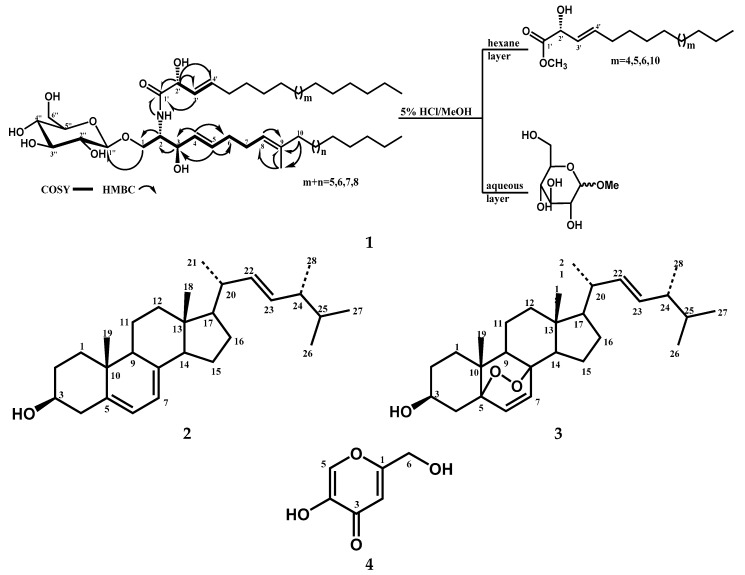
Structures of isolated compounds **1**–**4**.

Compound **1** (Figure 1) was isolated as a white solid, showing a single spot on Thin-layer chromatography (TLC). Compound **1** exhibited strong hydroxy (3343 cm^−1^) and amide absorption (1646, 1540 cm^−1^) bands in the IR spectrum (Figure S1).

The ^1^H-NMR spectrum (Figure S2) showed characteristic signals of an amide proton doublet at *δ*_H_ 8.33 (1H, d, *J* = 8.5 Hz), long methylene chain protons at *δ*_H_ 1.25, overlapped methyls at *δ*_H_ 0.85, and signals that corresponded to a monosaccharide (an anomeric proton at *δ*_H_ 4.91 (1H, d, *J* = 7.8 Hz), indicating a glycosphingolipid nucleus. The distinguishing peaks of 2-amino-1, 3,2′-triol of the hydrocarbon chain were detected at *δ*_H_ 4.77 (1H, m, H-2), 5.09 (1H, dd, *J* = 4.9, 0.7 Hz, H-2′), 4.75 (1H, m, H-1b), 4.24 (1H, dd, *J* = 10.4, 3.6 Hz, H-1a), and 4.77 (1H, m, H-3), and at *δ*_C_ 54.7 (C-2), 73.4 (C-2′), 70.03 (C-1), and 72.2 (C-3) in the NMR spectra.

The ^13^C-NMR spectrum (Figure S3) also revealed characteristic carbon peaks at *δ*_C_ 62.6 (CH_2_), 71.5 (CH), 75.1 (CH), 78.4 (CH), 78.5 (CH), and 105.6 (CH), indicative of the existence of sugar moiety. The ^1^H- and ^13^C-NMR spectra in the HSQC spectrum (Table 1, Figure S4) showed a disubstituted double bond at C-4 [*δ*_H_ 5.99 (1H,dd, *J* = 15.2, 5.2 Hz, H-4), *δ*_C_ 131.8 (CH, C-4), *δ*_H_ 5.96 (1H, dt, *J* = 15.5, 5.5 Hz, H-5), and *δ*_C_ 131.1 (CH, C-5)], an additional trisubstituted double bond [*δ*_H_ 5.25 (1H, brst, *J* = 7.8 Hz, H-8), *δ*_C_ 124.1 (CH, C-8), and *δ*_C_ 135.9 (C, C-9)] in long-chain base residues, and an extra disubstituted double bond [*δ*_H_ 6.11 (1H, dd, *J* = 15.3, 5.1 Hz, H-3′), *δ*_C_ 130.0 (CH, C-3′), *δ*_H_ 6.18 (1H, dt, *J* = 15.3, 6.4 Hz, H-4′), and *δ*_C_ 132.2 (CH, C-4′)] (Table 1) in fatty acid residues. The large vicinal coupling constant of olefinic protons at C-4 (*J*_H4–H5_ = 15.5 Hz) clearly indicated an E geometry for the double bond [36], which was further supported by the chemical shift of allylic carbons [*δ*_C_ 33.0 (C-6)] [37]. By the same manner, the geometry of the double bond at C-3′ was presumed to be E due to the large vicinal coupling constant of the olefinic protons (*J*_H3′–4′_ = 15.3 Hz) [36]. The geometry of the double bond at C-8 was presumed to be E due to the ^13^C-NMR signal of the methyl group attached to C-8 at *δ*_C_ 16.1, while that of a (Z)-C=C bond appeared at *δ*_C_ 22.7 [38]. The 2-amino-1,3-dioxigenated-4-ene moiety was confirmed by COSY correlations for H-1 through H-8 (Figure 1, Figure S5). The key HMBC correlations (Table 1, Figure S6), from H-1 to C-1″; from 2-NH to C-1′; from H-2 to C-1; from H-3 to C-2; from H-5 to C-3 and C-6; from H-8 to C-9; from H-10 to C-9; from CH_3_ at C-9; from CH_3_ at C-9 to C-8 and C-9; from H-2′ to C-1′, C-3′, and C-4′; and from H-3′ to C-1′, showed the connections C1-O-C1″ and C2-NH-C1′. Both connections were indicative of 1-*O*-glucopyranosyl-2-amido-3,2′-dihydroxy-9-methyl-4,8-diene groups (Figure 1). Furthermore, compound **1** was proposed to have normal-type side chain [37] because the terminal methyl signals in the ^13^C-NMR spectrum were detected at *δ*_C_ 14.2 (normal type) (Table 1, Figure S3).

The negative-ion FAB-MS spectrum [M − H]^−^ showed a succession of molecular ion peaks (Figure S7) at *m*/*z*: 710, 724, 738, and 752, with characteristic fragments at *m*/*z*: 310 and 268 indicating C-16 and C-19 long-chain bases. Therefore, the previously mentioned spectral data showed that compound **1** was assumed to be a molecular species of sphingosine-type cerebrosides with 2-hydroxy fatty acid.

The configuration of compound **1** was deduced to be (2*S*, 3*R*, 2′*R*), since the aforementioned ^13^C- NMR signals (C-1, C-2, C-3, C-2′) and ^1^H-NMR (H-2, H-3, H-2′), in addition to optical rotation value ${\left[\alpha \right]}_{D}^{19}$ = −7), were fitted with sphingosine-type cerebroside molecular species possessing (2*S*, 3*R*, 2′*R*) conformations [39].

The sugar moiety and length of the fatty acid residues were identified via methanolysis with methanolic hydrochloric acid of compound **1** followed by partition with *n*-hexane. After separating the two layers, the hexane layer gave a mixture of fatty acid methyl esters (FAMEs), while the aqueous layer afforded methylated sugar moiety. HPLC comparison between the methylated sugar against standard methylated sugars (glucose and galactose) confirmed that the sugar moiety was glucose (glucose *t*_R_ = 14.11 min, galactose *t*_R_ = 13.27). The anomeric proton-coupling constant at *δ*_H_ 4.91 (1H, d, *J* = 7.8 Hz) and the chemical shift of the anomeric carbon *δ*_C_ (105.6) confirmed the *β-*configuration of the glucopyranoside moiety (*α*-glucopyranoside: *J* = 3.7 Hz; δ_C_ 98.5) [36]. Analysis of the negative FAB-MS spectrum of the FAME mixture exhibited the presence of four components at *m*/*z*: 269, 283, 297, and 453 [M − H]^−^, which were considered as FAME-1, FAME-2, FAME-3, and FAME-4, indicating C-16, C-17, C-18, and C-22 FAMEs, respectively (Figure S8). Compound **1** (Figure 1) was first hydrolyzed, then the reaction mixture was extracted with hexane, and the hexane layer was concentrated to afford a mixture of the methyl esters of the α-hydroxy configuration fatty acids ${\left[\alpha \right]}_{D}^{19}$ = −5.0 (*c* 0.12, CHCl_3_) [39,40,41,42].

The method of Tanaka et al. [43] was used for determination of the absolute configuration of sugar moiety. Direct HPLC analysis of the sugar moiety reaction mixture exhibited a peak at *t*_R_ =18.68 min, which was matched with derivatives of D-glucose, confirming the absolute configuration of the sugar moiety (L-glucose *t*_R_ = 19.22 min). From previous data, the structure of compound **1** was identified as molecular species cerebroside, as shown in Figure 1. The name LAMA was assigned to compound **1**.

**Figure 2:**
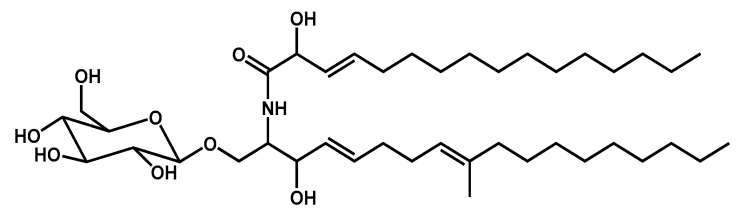
Structure of compound LAMA-1.

Due to the considerable importance of determining the molecular species composition of sphingolipids, isolation and structure elucidation of the cerebroside components in the molecular species of LAMA were conducted. By using reversed phase column, LAMA was fractionated to give LAMA-1. On the basis of the molecular mass of LAMA-1, *m*/*z* 748 [M + Na]^+^, 726 [M + H]^+^, 724 [M − H]^−^, and the characteristic fragments at *m*/*z* 562 corresponding to the aglycone part in addition to *m/z* 472 corresponding to glucosylated long chain base (Figures S7 and S9), the structure of this compound was considered resolved, as shown in Figure 2.

In addition to LAMA-1, the structures of other known compounds **2**–**4** (Figure 1) were elucidated by comparing their detailed NMR spectral data with those in the literature. These compounds were identified as ergosterol (**2**) [28,29,30], epidioxyergosterol (**3**), [30,31], and kojic acid (**4**) [32,33,34,35].

### 2.3. Cytotoxic Activity of Isolated Compounds ***1**–**4***

The isolated compounds **1**–**4** were evaluated for their cytotoxic activity against five selected human cancer cell lines (Table 2) using SRB assay. Compounds **2** and **3** displayed a promising cytotoxic profile against lung-cancer (A-549), prostate cancer (DU-145), breast adenocarcinoma (MCF-7), and hepatocellular carcinoma (HepG2) cell lines with IC_50_ values of 21.26, 19.3; 1.50, 6.10; 16.95, 13.6; and 2.89, 3.07 µM, respectively, while they were inactive against HeLa cells. Compounds **1** and **4** showed weak cytotoxic profiles against all cell lines under investigation (Table 2).

## 3. Materials and Methods

### 3.1. Biological Materials

**Figure 3:**
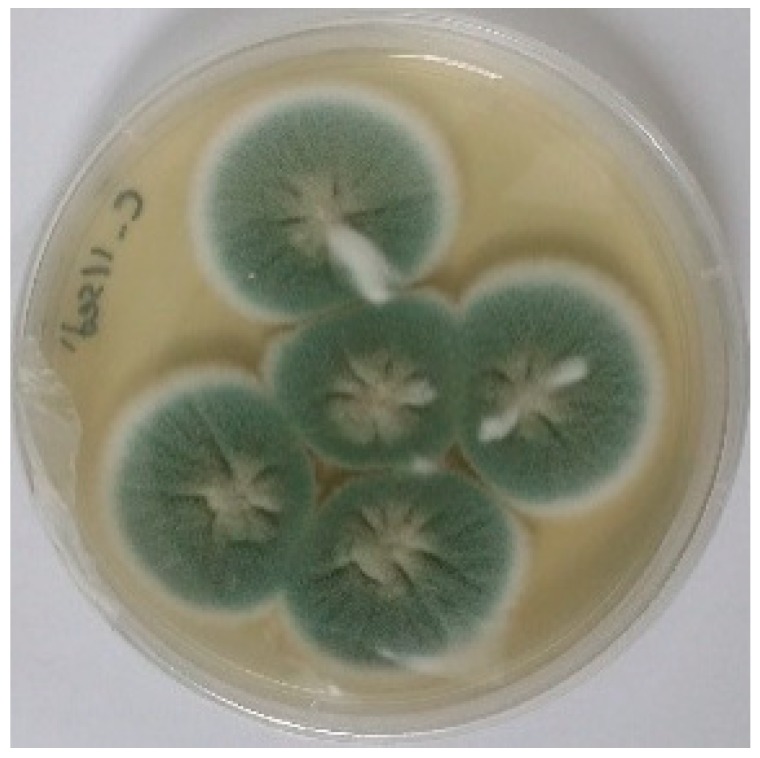
Morphological photo of the deep-sea-sediment-derived *Penicillium chrysogenum* strain S003.

The fungus *Penicillium chrysogenum* strain S003 (Figure 3) was cultured from deep-sea sediment from the Red Sea, and the fungal strain was identified based on a previously described method [26].

### 3.2. Fermentation and Extraction of Fungus Penicillium chrysogenum Strain S003

Fungus *Penicillium chrysogenum* strain S003 was cultured at 25 ℃ in 2 L Erlenmeyer flasks containing 500 mL of Czapek–Dox yeast liquid culture medium, composed of (NaNO_3_ 3.0 g/L, KCl 0.5 g/L, K_2_HPO_4_ 0.1 g/L, MgSO_4_·7H_2_O 0.5 g/L, FeSO_4_ 0.01 g/L, sucrose 30.0 g/L, yeast extract 5.0 g/L and NaCl 20.0 g/L). After cultivation for 30 days under static conditions, 15 L of the whole broth was filtrated using cheesecloth. Extraction of the broth with ethyl acetate was performed three times, and was further dried under vacuum to afford an ethyl acetate extract (1.4 g), while the mycelia were extracted three times with MeOH. The MeOH solution was partitioned with *n*-hexane, followed by evaporation to give a MeOH extract and an *n*-hexane extract (5 g). The MeOH extract was dissolved in water and partitioned with CHCl_3_, followed by evaporation under reduced pressure to produce an CHCl_3_ extract (2.8 g). The resulting extracts were subjected to further fractionation and separation to obtain the pure compounds.

### 3.3. Isolation and Purification of Compounds ***1**–**4***

The resulting CHCl_3_ extract (C-M-C, 2.8 g) from the previous section was chromatographed using CHCl_3_: MeOH (9.5:0.5~4:6) on SiO_2_ gel to yield 11 subfractions (C-M-C-1 to C-M-C-11). Fraction C-M-C-7 (325 mg) was subjected to a Sephadex LH-20 column and eluted with MeOH to afford 5 fractions (C-M-C-7-1 to C-M-C-7-5). Fraction C-M-C-7-1 (194 mg) was passed through a SiO_2_ gel column using CHCl_3_/MeOH (97:3 to 90:10) to afford two subfractions (C-M-C-7-1-1 to C-M-C-7-1-2). Fraction C-M-C-7-1-2 (80 mg) was chromatographed over a SiO_2_ gel column using CHCl_3_/MeOH (95:5) to yield phytoceramide molecular species compound (**1**) (30 mg). The name LAMA was assigned to compound **1**. Compound **1** was fractionated over reversed-phase column chromatography using H_2_O/MeOH (2:8 to 0.5:9.5) to give LAMA-1.

The EtOAc extract (C-E, 1.4 g) was eluted on Sephadex LH-20 column with MeOH/CHCl_3_ (1:1) to yield 10 subfractions (C-E-1 to C-E-10). Fraction C-E-4 (0.5 g) was passed through a SiO_2_ column with increasing EtOAc in *n*-hexane as eluent to obtain six fractions (C-E-4-1 to C-E-4-6). Fraction C-E-4-3 (195 mg) was subject to further chromatography using *n*-hexane: EtOAc (9.5:0.5 to 1:9) over a SiO_2_ gel column to give eight fractions (C-E-4-3-1 to C-E-4-3-8). Fraction C-E-4-3-2 (24 mg) was purified over a silica gel column using *n*-hexane/EtOAc (9.5:0.5 to 1:1) to get ergosterol (**2**) (3.1 mg) and epidioxyergosterol (**3**) (1.4 mg).

Chromatography for fraction C-E-6 (190 mg) over the SiO_2_ gel column using *n*-hexane/EtOAc (2:8 to 0:10) gradient elution produced seven subfractions (C-E-6-1 to C-E-6-7). Fraction C-E-6-4 (57 mg) was fractionated using MeOH/H_2_O (6:4) isocratic elution on reversed phase column, followed by gradient elution using *n*-hexane/EtOAc (2:8~0:10) on SiO_2_ gel column to obtain kojic acid (**4**) (20 mg).

### 3.4. Cytotoxicity of Compounds ***1**–**4***

The cytotoxic activity (Table 2) of the compounds on lung cancer A-549, cervical cancer HeLa, prostate cancer DU-145, hepatocellular carcinoma HepG2, and breast-adenocarcinoma MCF-7 cells was determined using an SRB assay [44,45], as mentioned in our study [46].

## 4. Conclusions

Chemical investigation of deep-sediment-derived *Penicillium chrysogenum* S003 yielded a cerebroside molecular species (**1**), and three known compounds: ergosterol (**2**), epidioxyergosterol (**3**), and kojic acid (**4**). The chemical structures of purified compounds **1**–**4** were characterized using spectroscopic studies and by comparison with available data in the literature. The cytotoxic activities of isolated compounds **1**–**4** against five human carcinoma cells were evaluated using an SRB assay. Compounds **2** and **3** displayed a promising cytotoxic profiles against lung cancer (A-549), prostate cancer (DU-145), breast adenocarcinoma (MCF-7), and hepatocellular carcinoma (HepG2) cell lines with IC_50_ values of 21.26, 19.3; 1.50, 6.10; 16.95, 13.6; and 2.89, 3.07 µM, respectively, while they were inactive against HeLa cells. Compounds **1** and **4** showed weak cytotoxic profiles against all cell lines under investigation.

## Figures and Tables

**Table 1:** NMR (Nuclear Magnetic Resonance) data of **1** (pyridine-*d*_5_).

Position	*δ*_C_	*δ*_H_ (m, *J* in Hz)	HMBC (H→C) ^a^
1	70.0, CH_2_	4.24 (dd, 10.4, 3.6) 4.75 (m)	C-1″
2	54.7, CH	4.77 (m)	C-1
3	72.2, CH	4.77 (m)	C-2
4	131.8, CH	5.99 (dd, 15.5, 5.2)	C-3, C-6
5	132.2, CH	5.96 (m), (dd, 15.5,5.5)	C-3, C-6
6	33.0, CH_2_	2.14 (m)	
7	32.1, CH_2_	2.14 (m)	
8	124.1, CH	5.25 (br t 7.8)	C-9
9	135.9, CH		
10	40.0, CH_2_	2.00 (t, 7.7)	C-9, C-9CH_3_
1′	173.8, C		
2′	73.4, CH	5.09 (dd, 4.9, 0.76)	C-1′, C-3′, C-4′
3′	130.0, CH	6.11 (dd, 15.3, 5.1)	C-1′
4′	132.2, CH	6.18 (dt, 15.3, 6.4)	
1″	105.6, CH	4.91 (d, 7.8 )	
2″	75.1, CH	4.02 (t, 6.6 )	
3″	78.4, CH	4.22 (m)	
4″	71.5, CH	4.22 (m)	
5″	78.5, CH	3.90 (m)	
6″	62.6, CH_2_	4.35 (dd, 11.8, 5.4) 4.51 (dd, 11.9, 2.5)	
CH_3_	14.2, CH_3_	0.85 (t, 7)	
9CH_3_	16.1, CH_3_	1.61 (s)	C-8, C-9
NH		8.33 (d, 8.5)	C-1′

^a^ HMBC correlations are from proton(s) stated to the indicated carbons.

**Table 2:** Cytotoxic activities of compounds **1**–**4** in µM against five selected human solid tumor cell lines (*n* = 3).

Cell Type	Cell Line	1	2	3	4
Lung cancer	A-549	>100	21.26 ± 0.18	19.30 ± 0.27	>100
Cervical cancer	HeLa	>100	>100	>100	>100
Prostate cancer	DU-145	>100	1.5 ± 0.03	6.1 ± 0.18	>100
Hepatocellular carcinoma	HepG2	>100	2.89 ± 0.23	3.07 ± 0.97	>100
Breast adenocarcinoma	MCF-7	>100	16.95 ± 0.53	13.6 ± 0.38	>100

## Supplementary Materials

The supplementary materials are available online at https://www.mdpi.com/2218-1989/10/2/75/s1. Figure S1. IR spectrum of compound LAMA; Figure S2. ^1^H-NMR spectrum of compound LAMA (pyridine-*d_5_*); Figure S3. ^13^C-NMR spectrum of compound LAMA (pyridine-*d_5_*); Figure S4. HSQC spectrum of compound LAMA (pyridine-*d_5_*); Figure S5. ^1^H-^1^H COSY spectrum of compound LAMA (pyridine-*d_5_*); Figure S6. HMBC spectrum of compound LAMA (pyridine-*d_5_*); Figure S7. FABMS spectrum of compound LAMA; Figure S8. FAM-FABMS spectrum of compound LAMA; Figure S9. FABMS spectrum of compound LAMA-1.
