# Supplementary material for: LAMA-1: A Cerebroside Isolated from the Deep-Sea-Derived Fungus Penicillium chrysogenum †
Source: Metabolites. 2020 Feb 20;10(2):75. doi: 10.3390/metabo10020075 (PMC7074484; doi:10.3390/metabo10020075)
Supplement: Supplementary file 1 [file metabolites-10-00075-s001.pdf]

LAMA-1: A New Cerebroside Isolated from the Deep-Sea Derived Fungus *Penicillium chrysogenum*

Samah O. Alshehri [1], Rania T. Malatani [1], Hanin A. Bogari [1], Ahmad O. Noor [1], Amany K. Ibrahim [2], Sameh S. Elhady [3,4,*], and Reda F. A. Abdelhameed [2,*]

[1] Department of Pharmacy Practice, Faculty of Pharmacy, King Abdulaziz University, Jeddah 21589, Saudi Arabia; salshehri1@kau.edu.sa (S.O.A.); rmalatani@kau.edu.sa (R.T.M.); hbogari@kau.edu.sa (H.A.B.); aonoor@kau.edu.sa (A.O.N.)

[2] Department of Pharmacognosy, Faculty of Pharmacy, Suez Canal University, Ismailia 41522, Egypt; am_kamal66@yahoo.com (A.K.I.); omarreda_70@yahoo.com (R.F.A.A.)

[3] Department of Natural Products, Faculty of Pharmacy, King Abdulaziz University, Jeddah 21589, Saudi Arabia; ssahmed@kau.edu.sa (S.S.E.)

[4] Department of Pharmacognosy, Faculty of Pharmacy, Port Said University, Port Said 42526, Egypt

* Correspondence: ssahmed@kau.edu.sa (S.S.E.); omarreda_70@yahoo.com (R.F.A.A.); Tel.: +966-544-512-552 (S.S.E), Tel.: +20-109-157-6797 (R.F.A.A.)

Additional Experimental Detail.

1) **Figure S1**. IR spectrum of compound **LAMA**
2) **Figure S2**. ^{1}H NMR spectrum of compound **LAMA** (pyridine-d_5)
3) **Figure S3**. ^{13}C NMR spectrum of compound **LAMA** (pyridine-d_5)
4) **Figure S4**. HSQC spectrum of compound **LAMA** (pyridine-d_5)
5) **Figure S5**. ^{1}H-^{1}H COSY spectrum of compound **LAMA** (pyridine-d_5)
6) **Figure S6**. HMBC spectrum of compound **LAMA** (pyridine-d_5)
7) **Figure S7**. FABMS spectrum of compound **LAMA**
8) **Figure S8**. FAM-FABMS of compound **LAMA**
9) **Figure S9**. FABMS of compound **LAMA-1**

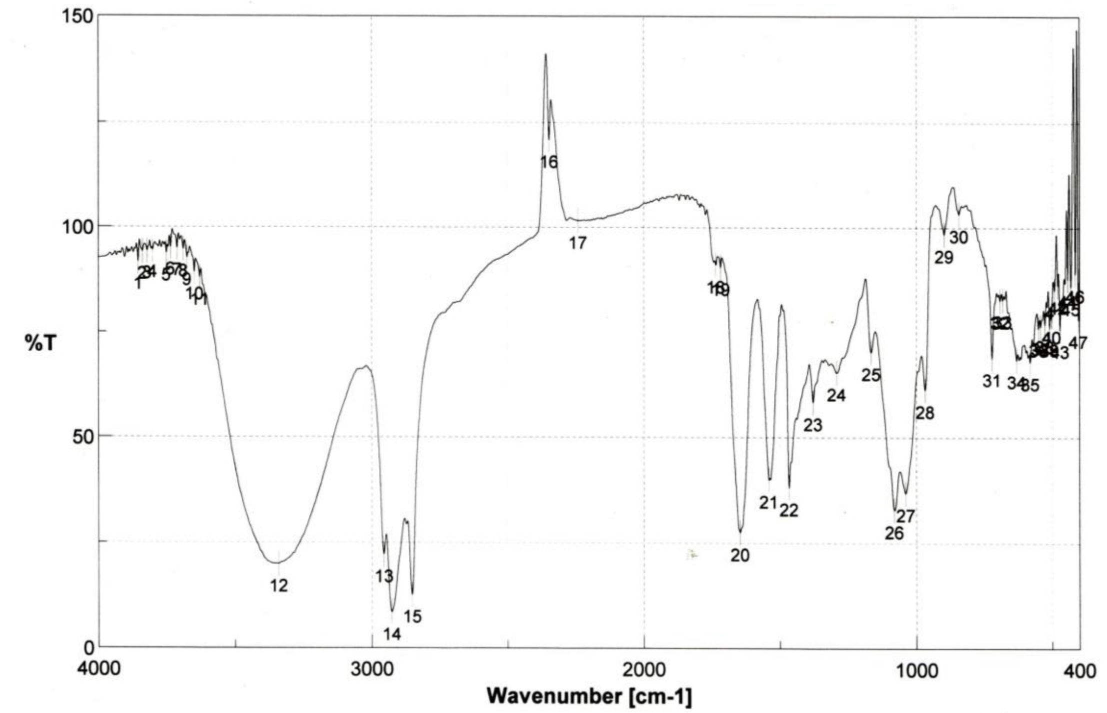

積算回数	Auto (222)
分解	4 cm-1
ゼロフィリング	ON
アポダイゼーション	Cosine
ゲイン	Auto (16)
スキャンスピード	Auto (2 mm/sec)
測定日時	2018/02/13 17:36
更新日時	2018/02/13 17:44
測定者	
ファイル名	Memory#4
サンプル名	20180119MGCuCl2_TFA_F1
コメント	

No.	cm-1	%T	No.	cm-1	%T	No.	cm-1	%T
1	3854.04	91.7768	2	3839.58	94.1968	3	3822.22	94.3343
4	3801.97	94.5471	5	3751.83	93.7976	6	3736.4	95.2495
7	3712.3	95.0242	8	3690.12	94.7185	9	3676.62	92.7506
10	3649.62	89.3198	11	3629.37	88.0441	12	3342.03	19.9744
13	2956.34	22.3118	14	2927.41	8.58859	15	2852.2	12.7336
16	2347.91	120.886	17	2241.84	101.601	18	1734.66	91.1273
19	1717.3	90.5828	20	1646.91	27.5001	21	1540.85	39.9378
22	1466.6	38.1224	23	1377.89	58.3835	24	1292.07	65.4312
25	1164.79	70.3237	26	1079.94	32.822	27	1038.48	36.7929
28	967.126	61.3574	29	896.737	98.4427	30	842.74	103.279
31	721.247	68.9926	32	692.32	82.7586	33	682.677	82.8951
34	631.573	68.4811	35	581.433	68.0113	36	551.542	76.0971
37	543.828	76.4867	38	527.436	77.0088	39	510.08	76.2062
40	502.366	79.2887	41	492.723	85.1862	42	480.188	86.3831
43	472.474	75.817	44	443.547	87.8302	45	431.977	86.0462
46	416.549	89.1081	47	404.978	78.1778			

Figure S1. IR spectrum of compound **LAMA**

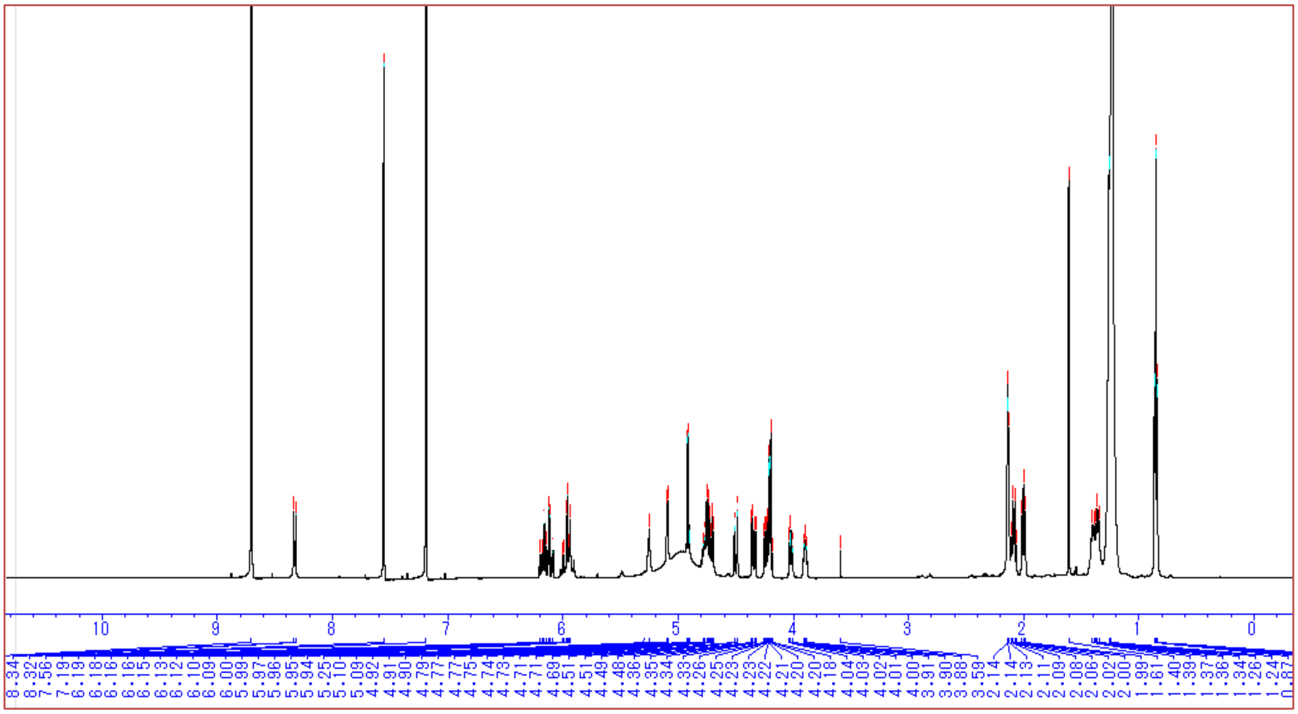

Figure S2. ^{1}H NMR spectrum of compound **LAMA** (pyridine-d_5)

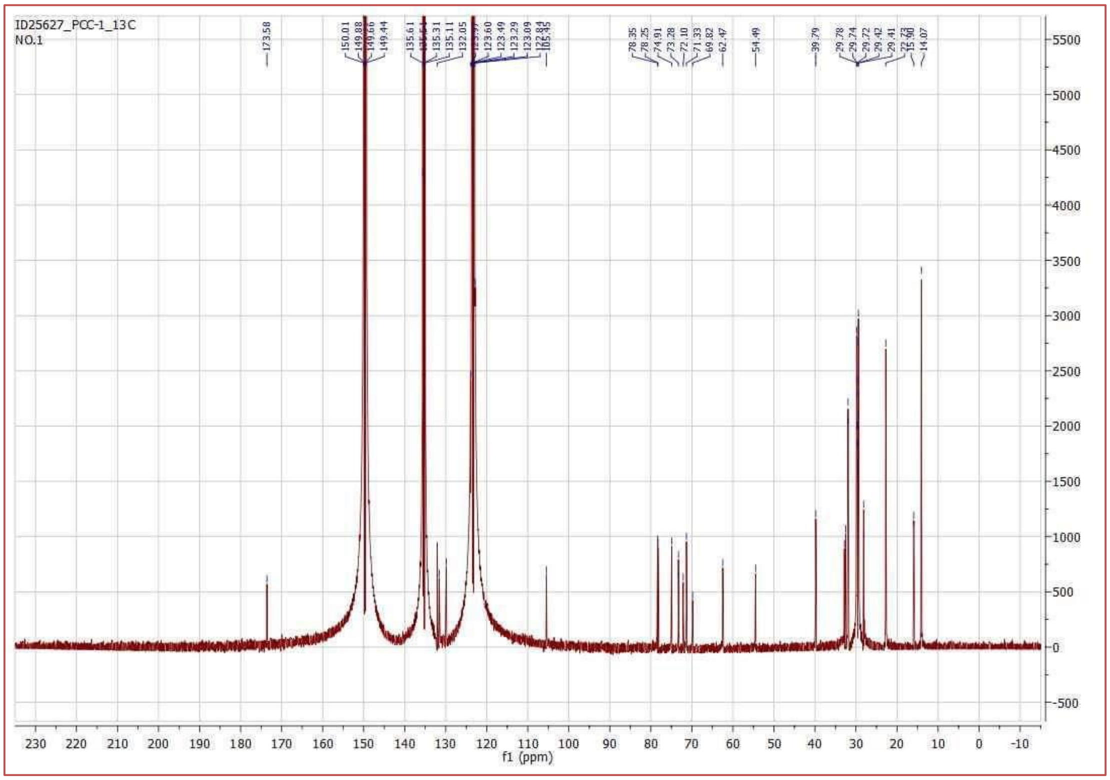

Figure S3. ^{13}C NMR spectrum of compound **LAMA** (pyridine-d_5)

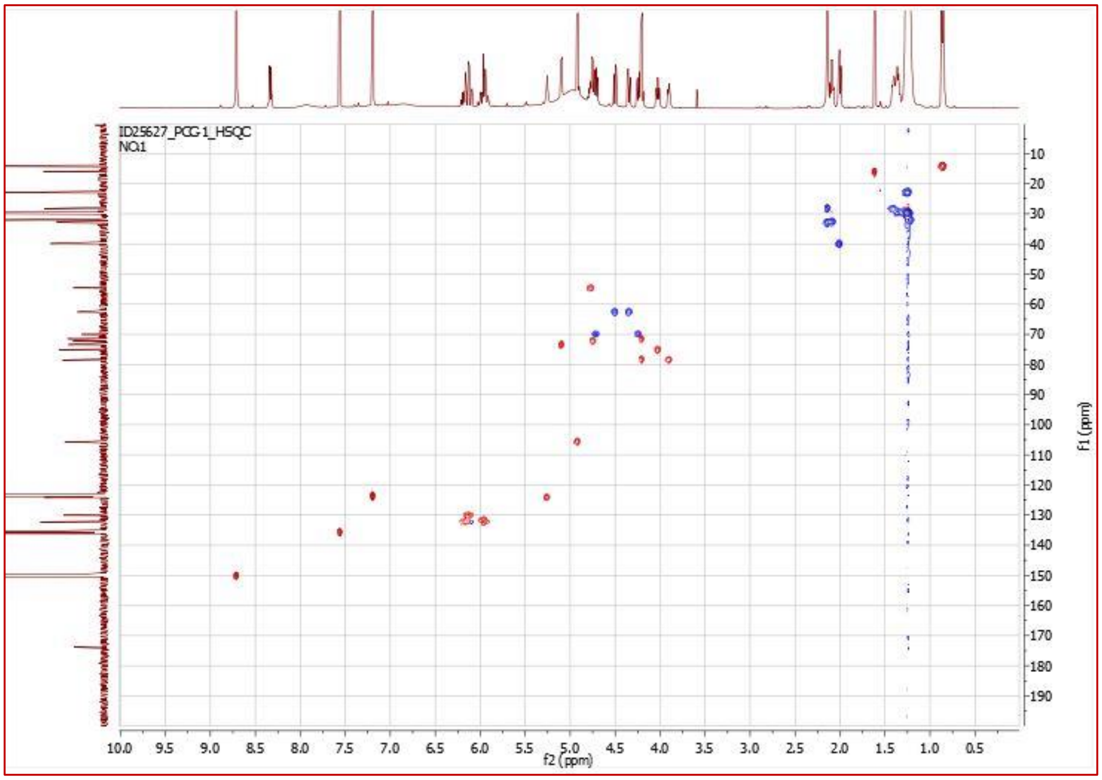

Figure S4. HSQC spectrum of compound **LAMA** (pyridine-d_5)

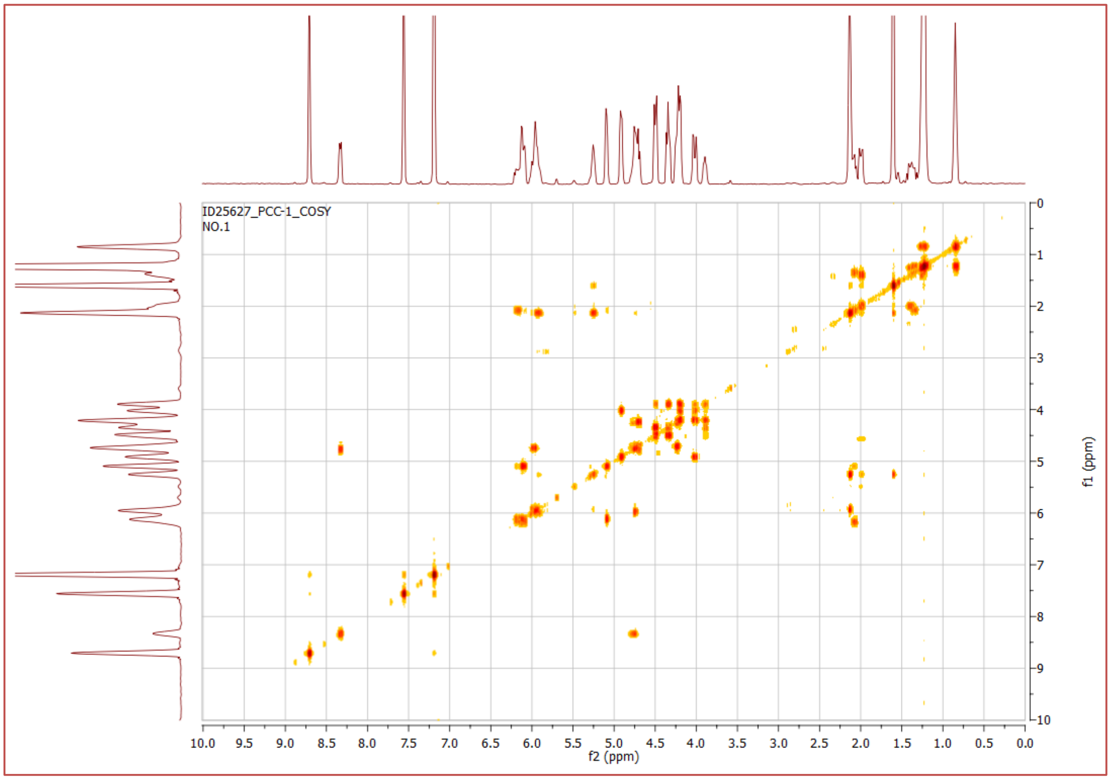

Figure S5. ^{1}H-^{1}H COSY spectrum of compound **LAMA** (pyridine-d_5)

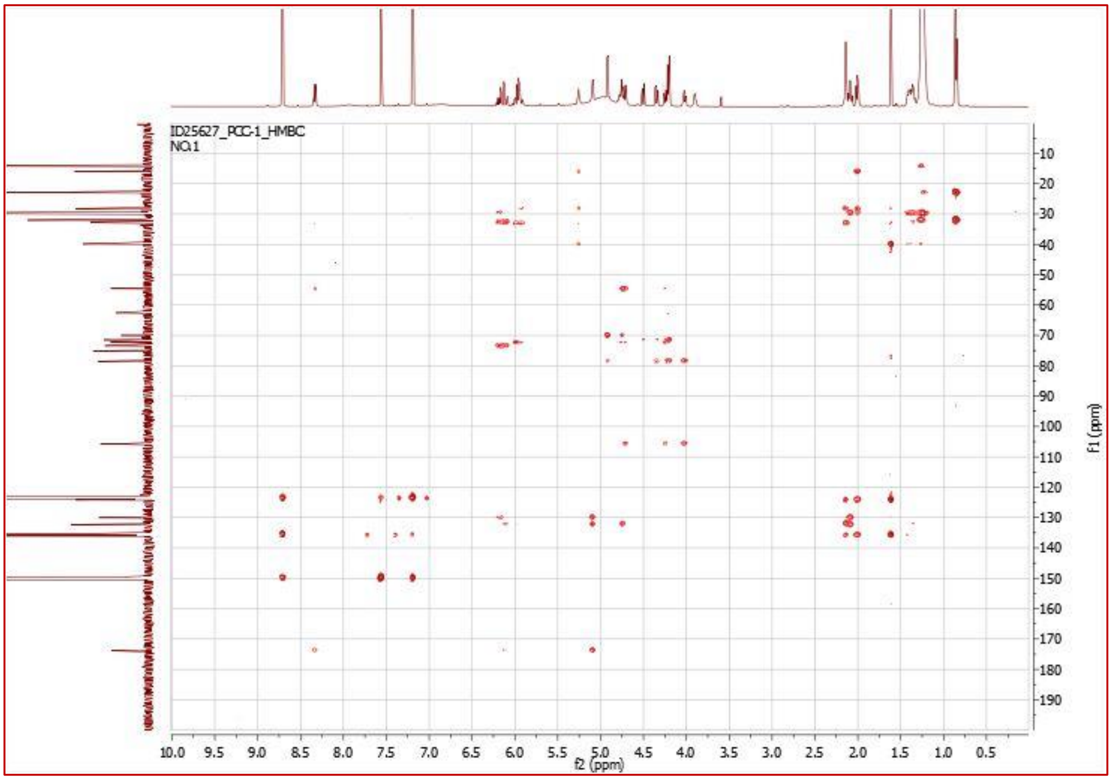

Figure S6. HMBC spectrum of compound **LAMA** (pyridine-d_5)

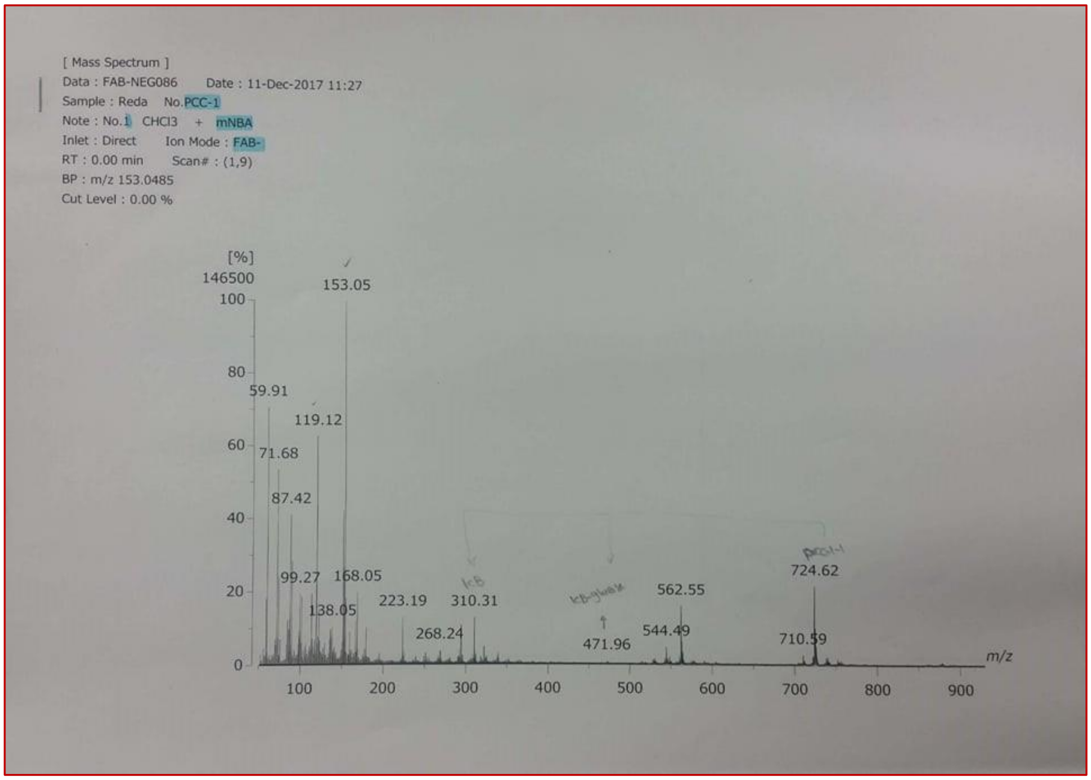

Figure S7. FABMS spectrum of compound **LAMA**

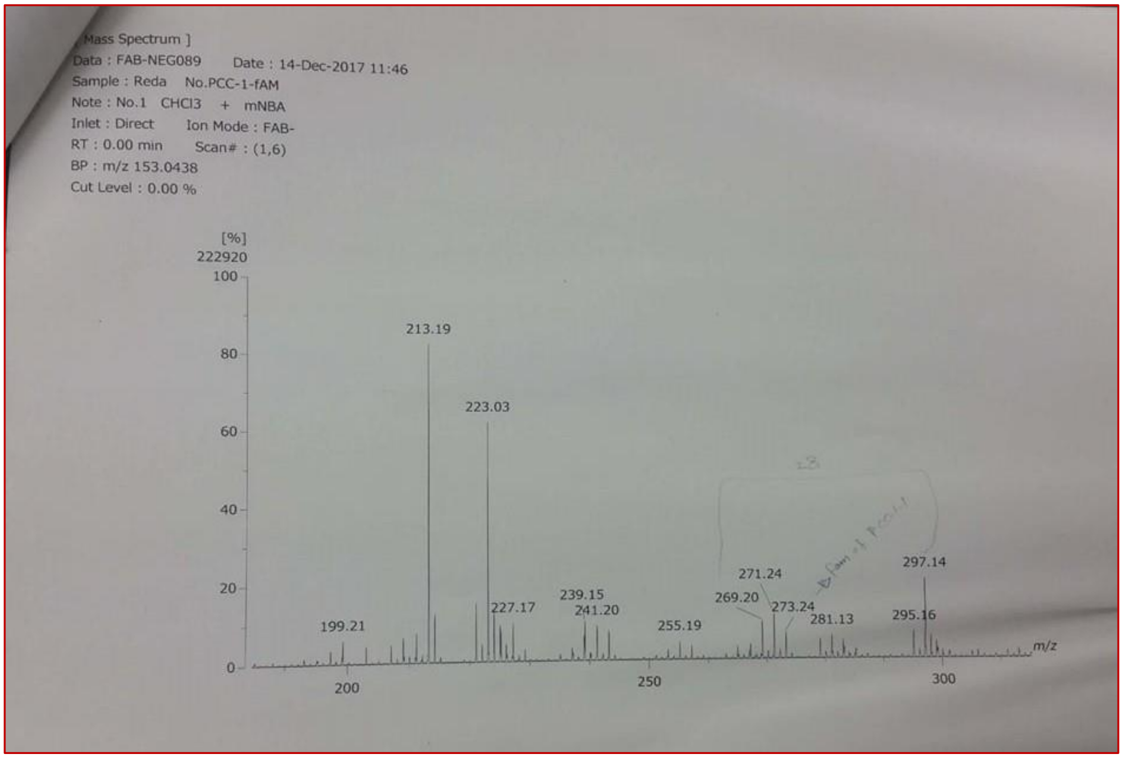

Figure S8. FAM-FABMS spectrum of compound **LAMA**

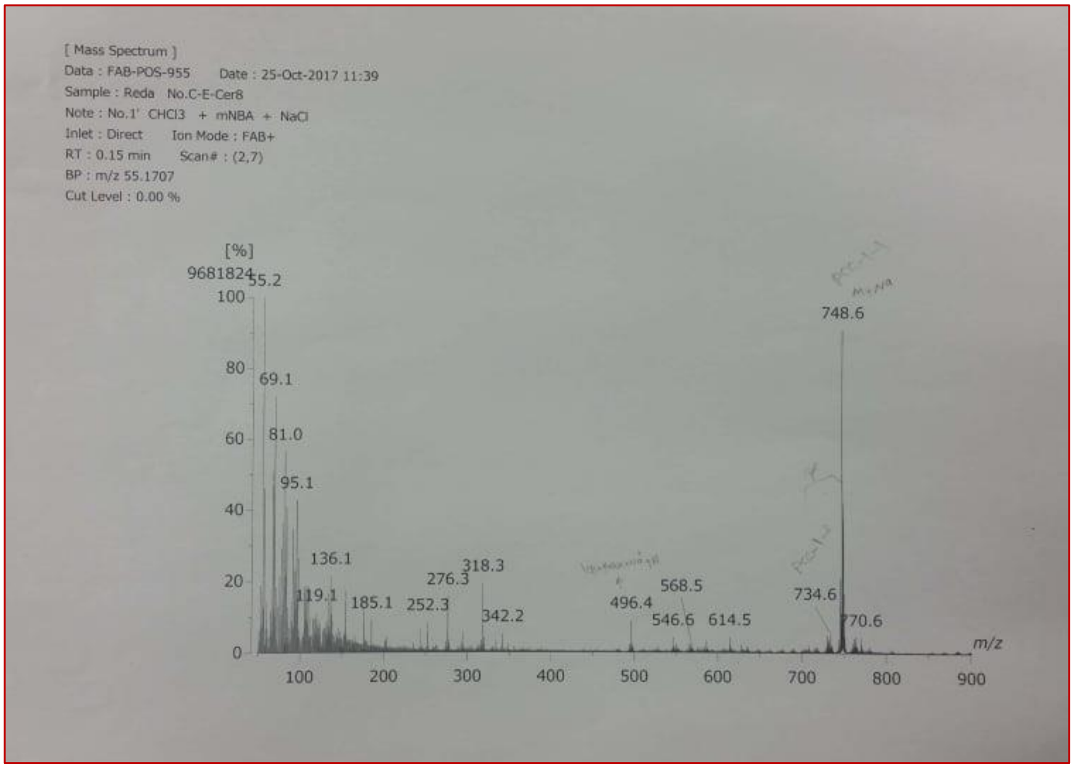

Figure S9. FABMS spectrum of compound **LAMA-1**
